# Continuous extracorporeal hyperoxygenation therapy reduces carbon monoxide half-life time in a carbon monoxide-poisoned pig model: a feasibility study

**DOI:** 10.1038/s41598-026-57491-5

**Published:** 2026-07-02

**Authors:** A. Fischbach, P. C. Schlanstein, M. F. Menne, C. Hoffmann, C. Lübke, H. Lüken, S. B. Wiegand, S. V. Jansen, U. Steinseifer, N. B. Steuer, R. Kopp

**Affiliations:** 1 Department of Intensive Care Medicine, Medical Faculty, RWTH Aachen University, Pauwelsstraße 30, 52074 Aachen, Germany; 2 Cardiovascular Engineering, Applied Medical Engineering, Medical Faculty, RWTH Aachen University, Forckenbeckstraße 55, 52074 Aachen, Germany; 3 Department of Anesthesiology and Intensive Care Medicine, Hannover Medical School, Carl-Neuberg-Str. 1, 30625 Hannover, Germany

**Keywords:** Carbon monoxide, Carbon monoxide poisoning, Extracorporeal membrane oxygenation, Hyperbaric oxygen therapy, Carbon monoxide elimination, Diseases, Medical research, Physiology

## Abstract

Carbon monoxide (CO) poisoning is a leading cause of toxic exposure-related morbidity and mortality worldwide. Although hyperbaric oxygen therapy increases CO elimination, its clinical use is limited by accessibility, side effects, and inconclusive long-term efficacy. We developed a cEHT system - a device for continuous extracorporeal hyperoxygenation therapy that enhances CO removal from blood without exposing patients to systemic hyperbaric conditions. The system was tested in vitro and in vivo in a porcine CO poisoning model. Carboxyhemoglobin (COHb) half-life, hemodynamics, hemolysis, and tissue hypoxia were evaluated. In vivo, the cEHT system significantly reduced COHb half-life by 53% compared to ventilation with 100% oxygen alone (27.0 ± 0.3 min vs. 57.6 ± 12.5 min; *p* = 0.01). Hemodynamic parameters, pulmonary artery pressure, and plasma-free Hb remained stable during treatment. Histological analyses showed reduced ischemic injury in heart and brain tissues in the cEHT group in contrast to the control group. The cEHT system enables effective and hemodynamically stable extracorporeal CO elimination. It may offer a promising therapy for CO poisoning when a pressure chamber is not promptly available. Further studies are needed to optimize system performance and assess clinical translation.

**Supplementary Information:**

The online version contains supplementary material available at 10.1038/s41598-026-57491-5.

## Introduction

Carbon monoxide (CO) is a colorless, odorless, and highly toxic gas produced during the incomplete combustion of carbon-containing fuels^1^ causing over 50,000 emergency department visits annually in the United States. It remains the leading cause of death from poisoning worldwide^2–5^, with mortality rate ranging from 1% to 3% ^6^. Long-term neurological sequelae affect up to 40% of survivors^7^, including cognitive impairment, motor dysfunction, and mood disorders^8^. Cardiovascular complications such as myocardial infarction, arrhythmias, and ventricular dysfunction further contribute to morbidity and mortality^9–12^, with US healthcare costs estimated at $1.3 billion per year^13^.

CO exerts its toxicity primarily through binding to heme-containing proteins, with approximately 250 times higher affinity to hemoglobin (Hb) than oxygen^14^. It not only impairs oxygen transport but also shifts the oxygen-Hb dissociation curve to the left, reducing oxygen release to tissues. Additionally, CO inhibits mitochondrial cytochrome c oxidase^15,16^, leading to cellular hypoxia and ATP depletion, particularly under hypoxic conditions^17^.

Oxygen therapy remains the mainstay of treatment. While normobaric oxygen reduces the COHb half-life to 1.5-2 h, hyperbaric oxygen therapy (HBO) shortens it to approximately 30 min^8,18–20^. However, limited availability of HBO facilities, the risk of potential adverse effects (e.g., barotrauma, oxygen toxicity), and inconclusive evidence regarding long-term efficacy represent significant limitations^21^. Moreover, CO toxicity does often not correlate with COHb levels, and low-dose chronic exposure may still result in severe neurological dysfunction due to oxidative and mitochondrial damage^22–24^.

Alternative strategies have emerged to address these limitations. Neuroglobin-based CO scavengers have shown promise in preclinical models by accelerating CO elimination and protecting mitochondrial function^25,26^. Another approach involves isocapnic hyperventilation, which enhances pulmonary CO clearance while maintaining normocapnia^27,28^. However, both methods face practical and translational challenges, such as technical complexity and lack of clinical validation.

In cases of CO poisoning with concurrent lung injury, such as from smoke inhalation, oxygen-based therapies become less effective due to impaired alveolar gas exchange^19^. Case reports have described the use of extracorporeal membrane oxygenation (ECMO) in patients with severe cardiopulmonary failure secondary to CO poisoning, offering an alternative route for systemic oxygenation and gradual CO removal^29–31^. To enhance extracorporeal CO clearance, a previous study introduced a light-assisted photo-ECMO device that significantly shortened COHb half-life through photodissociation in combination with elevated sweep gas pressures^32–34^.

Building on these concepts, we developed the extracorporeal hyperoxygenation therapy (EHT), an approach that uses high-pressure oxygenation within an external device to treat batches of CO-poisoned blood^35,36^. While effective in vitro and in large animal models, pressure fluctuations during device operation presented a major barrier to clinical translation.

To overcome these limitations, we developed a cEHT system (continuous extracorporeal hyperoxygenation therapy) for CO elimination. The system provides controlled extracorporeal hyperbaric oxygenation of blood in vivo without compromising hemodynamic stability. This study presents the design, in vitro validation, and in vivo feasibility of the cEHT system.

## Results

### In vitro CO elimination using the cEHT system

**Fig. 1:**
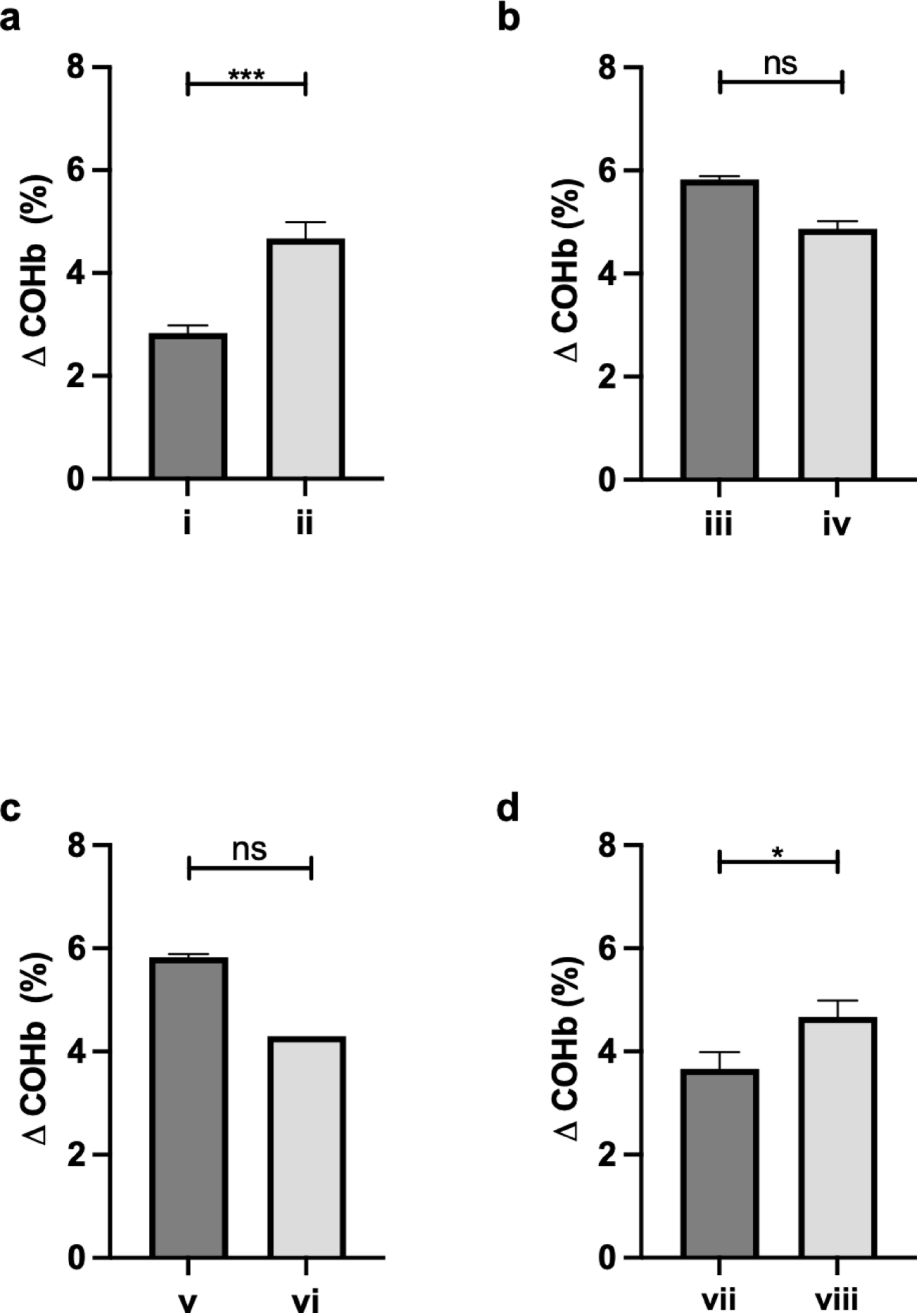
Effects of different parameters on CO elimination using the cEHT system in an in vitro single-pass setup. (**a**) Effect of different membrane surface areas on CO elimination. (**b**) Effect of different sweep gas composition through the first oxygenator of the cEHT system on CO elimination. (**c**) Effect of varying blood flow rates on CO elimination. (**d**) Effect of varying gas flow rates on CO elimination. The parameters for all setups (i-viii) shown on the x-axes in Fig. 1 are shown in Table 1. Three experiments were conducted for each setup.

To assess the effects of different parameters on CO elimination using the cEHT system, an in vitro single-pass model was used (Fig. 1). For each in vitro setup (i-viii), the membrane surface area of the first oxygenator (m²), the blood flow rate (L/min), the gas flow rate (L/min), the gas pressure (bar), and the gas composition of the gas flow are summarized in Table 1. In all in vitro experiments, the membrane surface area of the second oxygenator - the excess gas removal unit of the cEHT system - was 0.32 m². Three experiments were conducted for each setup.

**Table 1:** Experimental conditions of the setups (i-viii) shown in Fig. 1 for the in vitro experiments on CO elimination using the cEHT system.

Setup	Membrane surface area of the first oxygenator (m²)	Blood flow rate (mL/min)	Gas flow rate (L/min)	Gas pressure (bar)	Sweep gas composition (O_2_/CO_2_)
(i)	0.65	490	7	3.9	100% O₂
(ii)	1.9	490	7	3.9	100% O₂
(iii)	1.9	335	7	4.1	95% O₂ / 5% CO₂
(iv)	1.9	340	7	4.1	100% O₂
(v)	1.9	335	7	4.1	95% O₂ / 5% CO₂
(vi)	1.9	500	7	4.1	95% O₂ / 5% CO₂
(vii)	1.9	460	7	3.9	100% O₂
(viii)	1.9	500	5	2.9	100% O₂

The results demonstrated that a membrane surface area of 1.9 m² in the first oxygenator of the cEHT system resulted in a significantly greater reduction in COHb following a single blood pass through the system compared to an oxygenator with a smaller membrane surface area of 0.65 m² (2.8 ± 0.2% vs. 4.7 ± 0.3%; *p* < 0.001; Fig. 1a).

No significant difference in CO elimination was observed when comparing sweep gas compositions of 95% O₂ / 5% CO₂ versus 100% oxygen (Fig. 1b). Likewise, varying the blood flow rate between 335 mL/min and 500 mL/min did not result in a statistically significant difference in COHb reduction (Fig. 1c). However, a higher gas pressure in the first oxygenator (3.9 bar vs. 2.9 bar) resulted in a significantly greater elimination of CO (3.7% ± 0.3% vs. 4.7% ± 0.3%; *p* = 0.019).

### In vivo CO elimination using the cEHT system in a CO-poisoned pig model

**Fig. 2:**
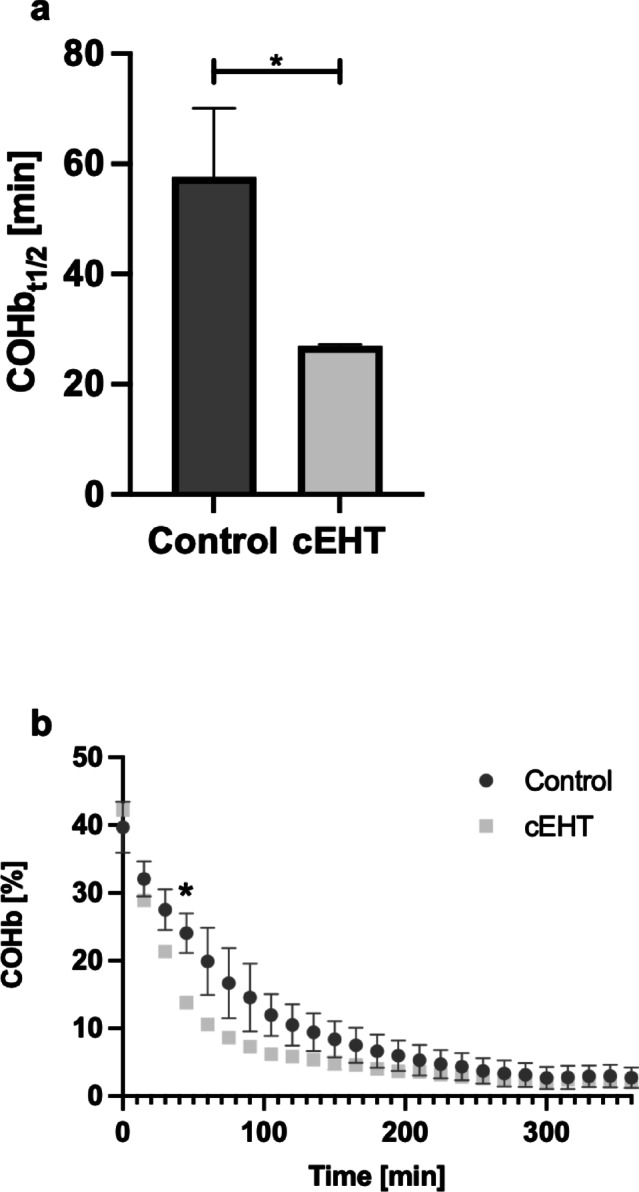
CO elimination in a porcine model using 100% oxygen alone (control; *n* = 3) or in combination with the cEHT system (*n* = 3). (**a**) COHb half-life (COHb t_₁/₂_); (**b**) COHb (%) over time (min) from the beginning of the recovery phase.

To evaluate whether treatment with the cEHT system significantly accelerates in vivo CO elimination, CO-poisoned pigs were treated either with 100% oxygen alone (control group) or in combination with the cEHT system. In animals treated with 100% oxygen alone, the COHb half-life was 57.6 ± 12.5 min. In contrast, animals of the cEHT group showed a significantly reduced COHb half-life of 27.0 ± 0.3 min (*p* = 0.01; Fig. 2a).

The mean values of COHb did not differ significantly between groups until 45 min after the start of treatment. At this time point, a significant difference was observed between the Control group (23.3% [range: 21.6-27.3%]) and the cEHT group (13.6% [range: 13.4-14.4%]); *p* = 0.0231; Fig. 2b.

**Fig. 3:**
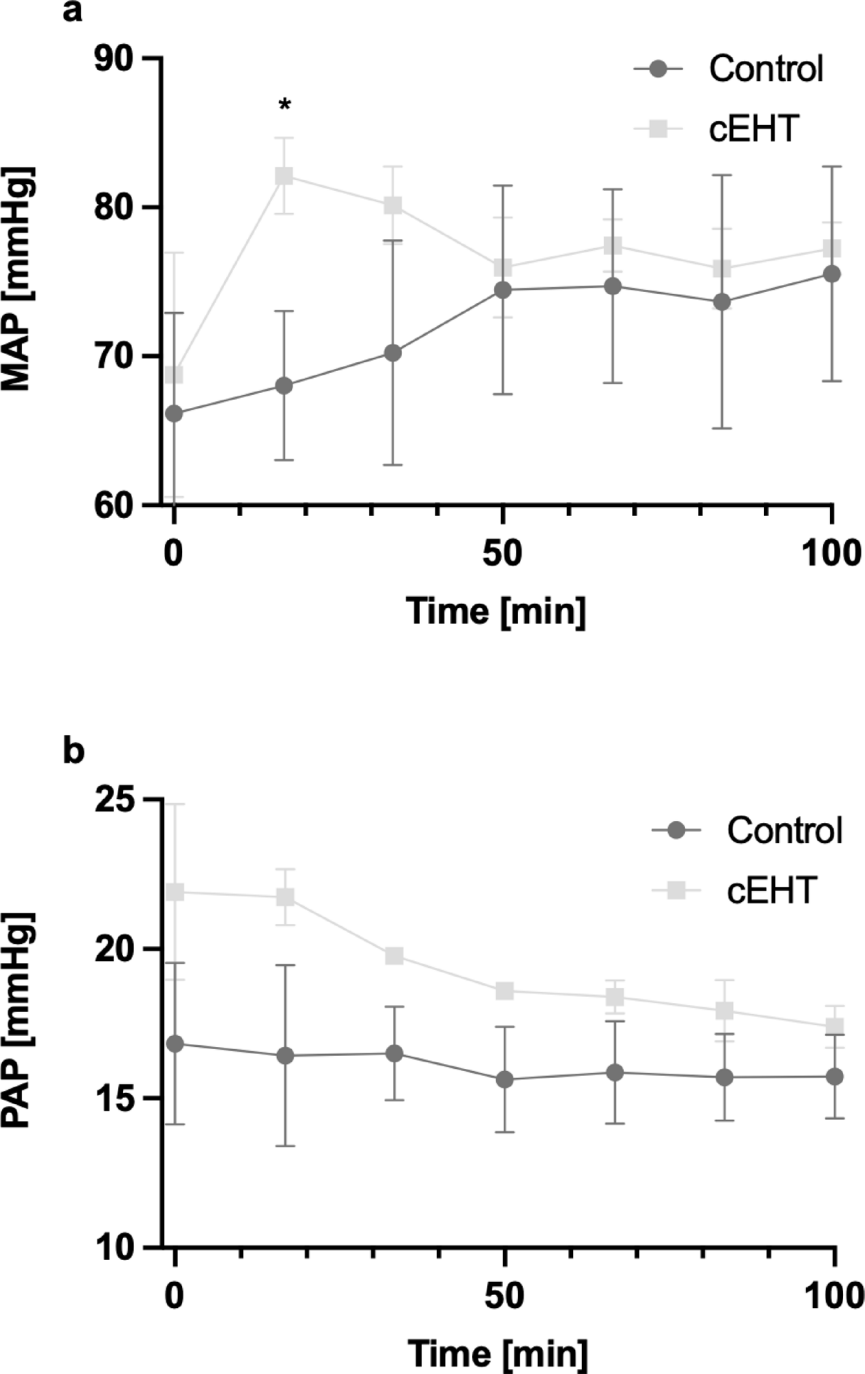
(**a**) Mean arterial pressure (MAP, mmHg) and (**b**) pulmonary arterial pressure (PAP, mmHg) during treatment with either 100% oxygen alone (control group; *n* = 3) or in combination with the cEHT system (*n* = 3).

To assess whether the cEHT induces blood pressure fluctuations, mean arterial pressure (MAP) values were compared between CO-poisoned pigs treated with 100% oxygen alone and those receiving additional treatment with the cEHT system. No significant differences in MAP were observed between the two treatment groups throughout the therapy, except at t = 16.7 min after the start of treatment, where MAP was significantly higher in the cEHT group compared to the control group (82.0 ± 2.4 mmHg vs. 68.0 ± 5.0 mmHg; *p* = 0.023) (Fig. 3a).

To evaluate whether a treatment with the cEHT system is associated with an elevated pulmonary arterial pressure (PAP), values were compared between both treatment groups. As shown in Fig. 3b, no significant differences in PAP were observed between animals treated with 100% oxygen alone and those receiving additional cEHT.

**Fig. 4:**
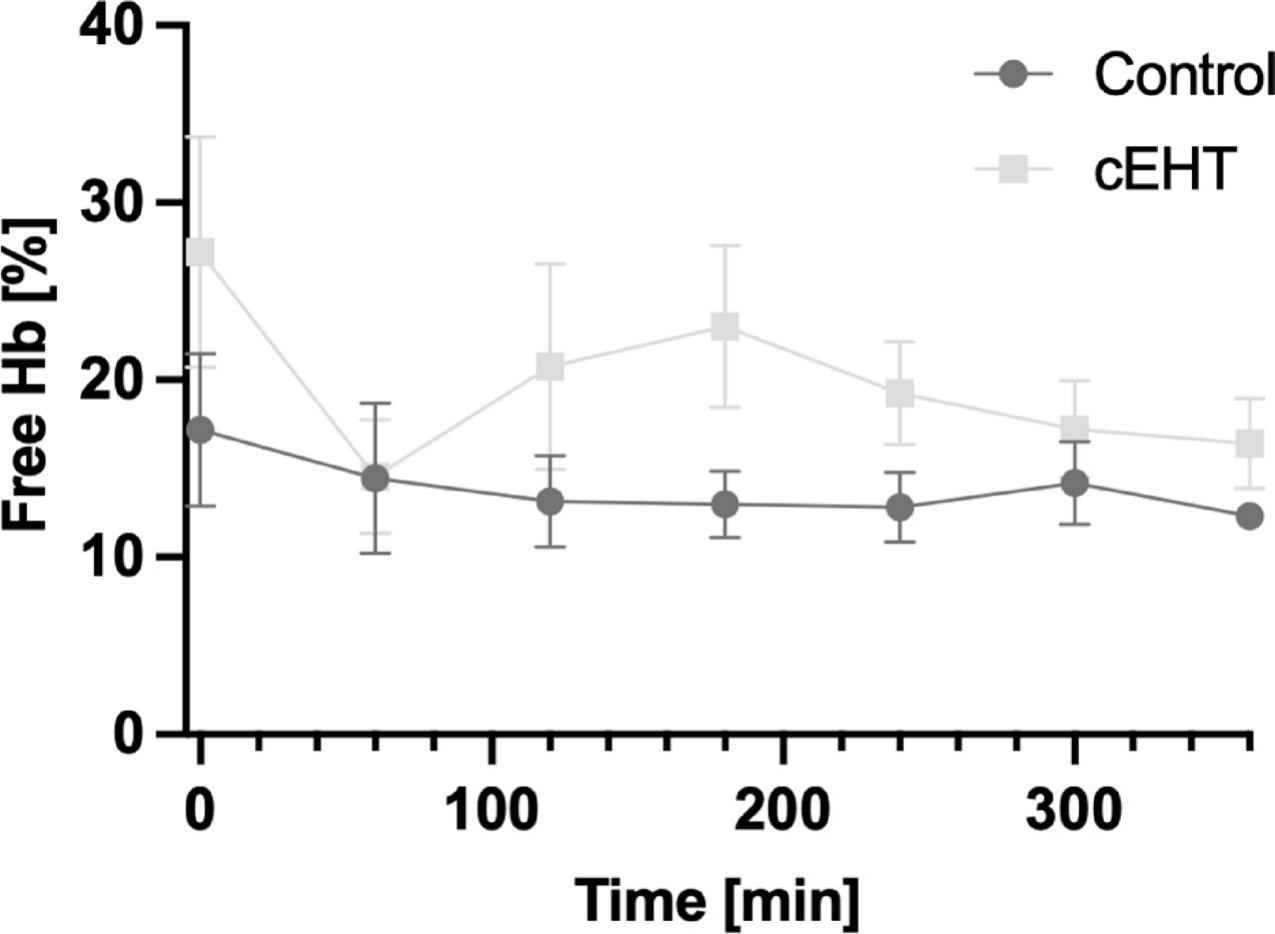
Concentration of plasma-free Hb (pfHb) during treatment with either 100% oxygen alone (control group; *n* = 3) or in combination with cEHT (*n* = 3).

To determine whether cEHT is associated with increased hemolysis, plasma-free Hb (pfHb) concentrations were measured during treatment of CO-poisoned pigs with either 100% oxygen alone or in combination with the cEHT system (Fig. 4). No significant differences in pfHb levels were observed between the two treatment groups. Additionally, pfHb concentrations remained consistently below 30 mg/dL throughout the treatment.

To evaluate whether treatment with the cEHT system reduces tissue ischemia in CO-poisoned pigs compared to standard therapy with 100% oxygen alone, histological samples from the heart and brain were collected at the end of the experiment.

**Fig. 5:**
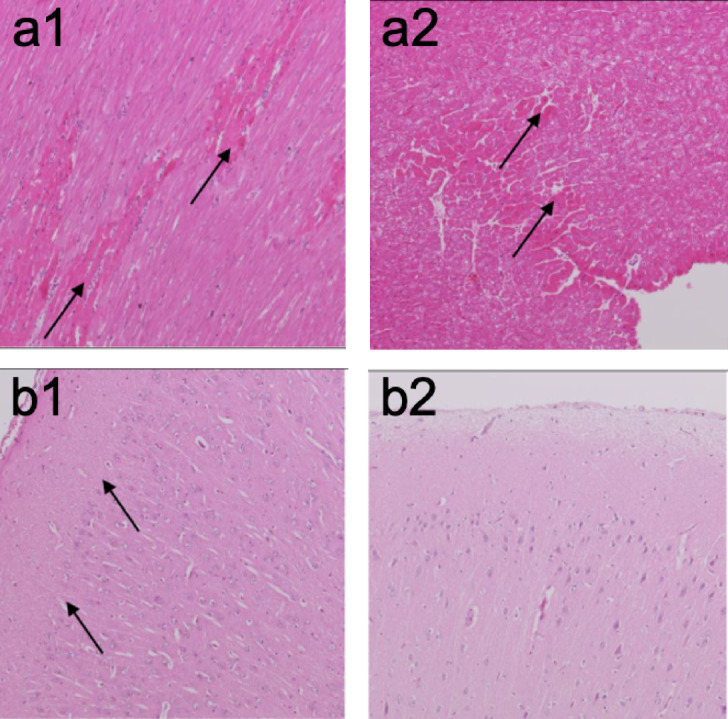
(**a**) Representative histological overview of cardiac tissue following treatment with 100% oxygen alone (a1) versus additional therapy using the cEHT system (a2). (**b**) Representative histological overview of brain tissue following treatment with 100% oxygen alone (b1) and additional cEHT (b2). Histology was performed on the Control group (*n* = 1) and the cEHT group (*n* = 3) for both heart and brain tissue.

In animals treated with 100% oxygen alone, focal areas of ischemia were observed microscopically in both cardiac and cerebral tissues (Fig. 5a1 and 5b1). In contrast, animals treated with the cEHT system showed no clear histological evidence of ischemic lesions in either the myocardium (Fig. 5a2) or brain tissue (Fig. 5b2).

To ensure comparable experimental conditions, systemic physiological parameters were monitored throughout the study. Systemic arterial pO_2_ levels remained comparable between both groups (*p* ≥ 0.10), with median values of 205.3 [range: 82.4-223.3] mmHg in the control group and 206.7 [range: 71.7-223.3] mmHg in the cEHT group (Supplementary Figure S2a). Similarly, arterial pCO_2_ levels showed no significant differences at any interval, remaining within physiological limits in both the control (37.2 [range: 35.2-38.2] mmHg) and cEHT groups (39.2 [range: 35.3-40.8] mmHg), which confirms consistent ventilatory management (Supplementary Figure S2b). Furthermore, no significant metabolic derangement was observed; median lactate levels were 0.8 [range: 0.6-1.3] mmol/L in the control group and 1.0 [range: 0.7-2.3] mmol/L in the cEHT group (Supplementary Figure S2c).

## Discussion

This study assessed the effect of cEHT on CO elimination under in vitro and in vivo conditions. In the in vitro experiments, a membrane surface area of 1.9 m² in the first oxygenator resulted in a significantly greater reduction in COHb compared to a smaller membrane surface. Variations in sweep gas composition (95% O₂ / 5% CO₂ vs. 100% oxygen), blood flow rate (335 mL/min vs. 500 mL/min), and gas flow rate (5 L/min vs. 7 L/min) did not lead to significant differences in COHb reduction.

In vivo, CO-poisoned animals treated with the cEHT system showed a significant shorter COHb half-life compared to animals receiving 100% oxygen alone. Mean arterial and pulmonary arterial pressures remained comparable between both groups, except for a single time point with a transient increase in MAP in the cEHT group. No significant differences in plasma-free Hb levels were observed. Histological analysis indicated fewer ischemic changes in myocardial and brain tissue in animals treated with the cEHT system.

The in vitro experiments demonstrated that increasing the membrane surface area significantly enhances CO elimination by increasing blood exposure to hyperbaric oxygen, thereby improving gas exchange efficiency.

In contrast, neither different blood flow rates nor gas flow rates significantly influenced CO elimination within the tested parameters (blood flow rate: 335 mL/min vs. 500 mL/min; gas flow rate: 5 L/min vs. 7 L/min). This lack of a clear effect may be explained by two opposing mechanisms: While higher blood flow rates could increase CO elimination by increasing the volume of blood exposed to the oxygen-rich environment, a reduced blood flow rate increases the exposure time of CO-poisoned blood within the blood compartment, which facilitates CO elimination. These opposing effects may have offset one another, resulting in similar CO elimination across the tested conditions. Nevertheless, variations beyond the tested blood flow range might result in changes in CO removal performance. However, higher blood flow rates require the use of larger vascular cannulas, thereby increasing invasiveness. As the system is intended primarily for preclinical or emergency use, future optimizations must carefully weigh therapeutic efficacy against the invasiveness of the system. Similarly, while no significant effect of different gas flow rate was observed within the tested range, different gas flow rates than tested in this study may produce different results. Therefore, future investigations should explore an expanded range of blood and gas flow rates to identify optimal parameters that maximize CO elimination while minimizing associated risks.

No significant differences in CO elimination were observed between different sweep gas compositions (95% O₂ + 5% CO₂ vs. 100% oxygen). To minimize the risk of respiratory alkalosis caused by prolonged exposure to 100% oxygen and to preserve physiological acid-base balance, 5% CO₂ was added to the sweep gas flow in subsequent experiments.

In vivo, the COHb half-life under treatment with the cEHT system was 27.0 ± 0.3 min, representing a reduction of approximately 53% compared to ventilation with 100% oxygen via tracheal tube (57.6 ± 12.5 min). These results were obtained in animals with a mean body weight of 79.2 ± 6.2 kg and an estimated blood volume of 6.3 L (based on 8% of total body weight)^37^.

In a previous study, we developed a bubble oxygenator system in which blood was withdrawn, exposed to hyperbaric oxygen, and subsequently re-infused into CO-poisoned animals^36^. That system achieved a comparable in vivo COHb half-life of 29.77 min. However, the cEHT system described in this study represents a significant improvement in maintaining hemodynamic stability, as both mean arterial pressure and pulmonary arterial pressure remained stable during the entire course of treatment. In contrast, the previously described extracorporeal hyperoxygenation therapy (EHT)^35,36^ was associated with elevated and fluctuating pulmonary arterial pressure, likely due to periodic volume shifts caused by the system’s mode of operation. Furthermore, the intermittent blood withdrawal and re-infusion in the bubble oxygenator led to transient reductions in MAP.

Another previously evaluated system, the photo-ECMO device^34^, achieved a 36% improvement in CO elimination compared to its control group. However, differences between studies regarding animal weight and thus blood volume must be considered. The animals in the photo-ECMO study had an average body weight of 45.3 ± 1.3 kg, corresponding to an estimated blood volume of approximately 3.6 L - about 43% less than in the present study.

Interestingly, in the cEHT group, COHb concentrations at individual time points were not significantly different from the control group (100% oxygen ventilation), except at 16.7 min after initiation of therapy. Nevertheless, the overall COHb half-life was significantly shorter when the cEHT system was used. This discrepancy may be explained by the nonlinear kinetics of CO elimination^38^: early changes in COHb concentration may appear modest, yet the cumulative effect over time results in a substantially accelerated decline. Small initial differences can thus result in greater effects as therapy progresses.

In our study, systemic arterial pO_2_ levels remained at approximately 200 mmHg. However, these levels must be compared to the oxygen tension within the cEHT circuit, where the therapeutic displacement of CO occurs. Although the system operated at a hyperbaric pressure of 4.6 bar, direct measurement of the pO_2_ within the circuit was not feasible as the levels exceeded the detection limits of standard blood gas analyzers. However, the oxygen tension within the treatment unit can be estimated using Henry’s Law^7,39^. At a pressure of 4.6 bar, the calculated oxygen tension reaches approximately 3290 mmHg, providing a massive partial pressure gradient that facilitates rapid CO elimination observed in the cEHT group.

For comparison, standard hyperbaric oxygen therapy typically operates at 2.5 to 3.0 ATA^40^, reaching systemic levels of roughly 1500 to 2000 mmHg. While blood within the cEHT circuit is exposed to high local oxygen partial pressures, systemic arterial pO_2_ remains at significantly lower, safer levels.

Given that the long-term morbidity of CO intoxication is mainly due to cardiac and neurological sequelae^41^, we also assessed histological sections for ischemic changes.

In our study, myocardial and cerebral tissue from animals treated with the cEHT system showed fewer signs of ischemia compared to the control group. This may suggest that more efficient CO elimination could positively impact long-term cardiac and cerebral outcomes. Longer-term studies are required to assess potential delayed cardiac and neurological damage. An important potential application of the cEHT system may be the treatment of CO-poisoned patients with concurrent acute lung injury. In such cases - often resulting from smoke inhalation, thermal damage, or exposure to toxic gases like NO₂ and hydrogen chloride - alveolar gas exchange is severely impaired^42–45^. As a result, the effectiveness of conventional oxygen therapy, including 100% oxygen ventilation, is significantly reduced. Under these conditions, pulmonary elimination of CO is compromised, delaying CO elimination and increasing the risk of systemic hypoxia and neurological injury. By enabling extracorporeal CO removal independent of pulmonary function, the cEHT system may provide a significant therapeutic advantage for this patient population. Future studies should therefore evaluate its efficacy specifically in the setting of CO poisoning complicated by lung injury. Beyond its application in CO poisoning, the cEHT system may also be beneficial in conditions characterized by impaired oxygenation, such as acute respiratory distress syndrome (ARDS)^46^, given its ability for efficient CO₂ removal and oxygenation.

Currently, beyond the administration of normobaric or hyperbaric 100% oxygen, only experimental strategies are available for the treatment of CO intoxication.

Neuroglobin-based CO scavengers like Ngb-H64Q-CCC have a high affinity for CO ^25,26^. They bind CO from Hb and mitochondria, and the resulting complex is subsequently excreted through the kidneys. In small animal studies, CO clearance was significantly increased. However, this approach has not yet been tested in humans and still faces challenges such as protein stability, dosing, and high production and storage costs.

Another approach is isocapnic hyperventilation^27,28^, in which the respiratory rate is increased to increase CO elimination, while CO₂ is simultaneously supplemented to prevent hypocapnia. This method has been shown to significantly reduce COHb half-life and shows CO elimination rates comparable to HBO therapy. However, its application is technically difficult and may cause discomfort in conscious patients due to the increased ventilatory rates.

The present study has several limitations.

First, in the in vitro setup, only a limited range of blood and gas flow rates was tested, given the feasibility-focused design of the present study and associated resource constraints. Future experiments should include a broader range of blood flow rates and gas flow rates to identify optimal parameters for maximizing CO elimination while minimizing device invasiveness and risk.

Second, the in vivo experiments were limited by the absence of an additional control group in which the first oxygenator would be exposed to normobaric instead of hyperbaric oxygen. Comparing hyperbaric and normobaric oxygen ventilation within the same circuit could help determine the impact of hyperbaric pressure on CO clearance.

Third, the number of animals was limited. As this was a pilot study to test the technical feasibility and safety of the cEHT system, a formal power analysis was not performed.Therefore, these results should be considered preliminary. Future research with larger sample sizes is needed to confirm our findings and provide a more robust statistical evaluation.

Fourth, to allow a more accurate assessment of therapeutic efficacy, future studies should include measurements of exhaled CO from the animals and quantify the CO eliminated through the cEHT system - similar to the approach taken in a previous study^34^.

Fifth, long-term effects of CO intoxication, especially cardiac and neurological outcomes, were not evaluated. Histological damage from CO is known to manifest 3-5 days post-exposure, particularly as hemorrhagic necrosis in the basal ganglia and subendocardial regions. To reliably assess these outcomes, follow-up periods of at least two weeks should be included into future studies, along with an increased number of animals per group to improve statistical power.

Lastly, the cEHT system will need further design optimization to enable its use in ambulances or in emergency rooms.

## Conclusion

In summary, this study demonstrated that extracorporeal hyperoxygenation using the cEHT system is a feasible and efficient method for CO elimination in a large animal model. Compared to conventional oxygen ventilation, the cEHT significantly reduced COHb half-life by 53% without causing hemodynamic instability or increased hemolysis. Further studies are needed to optimize the device, evaluate long-term outcomes, and assess clinical applicability.

## Materials and methods

**Fig. 6:**
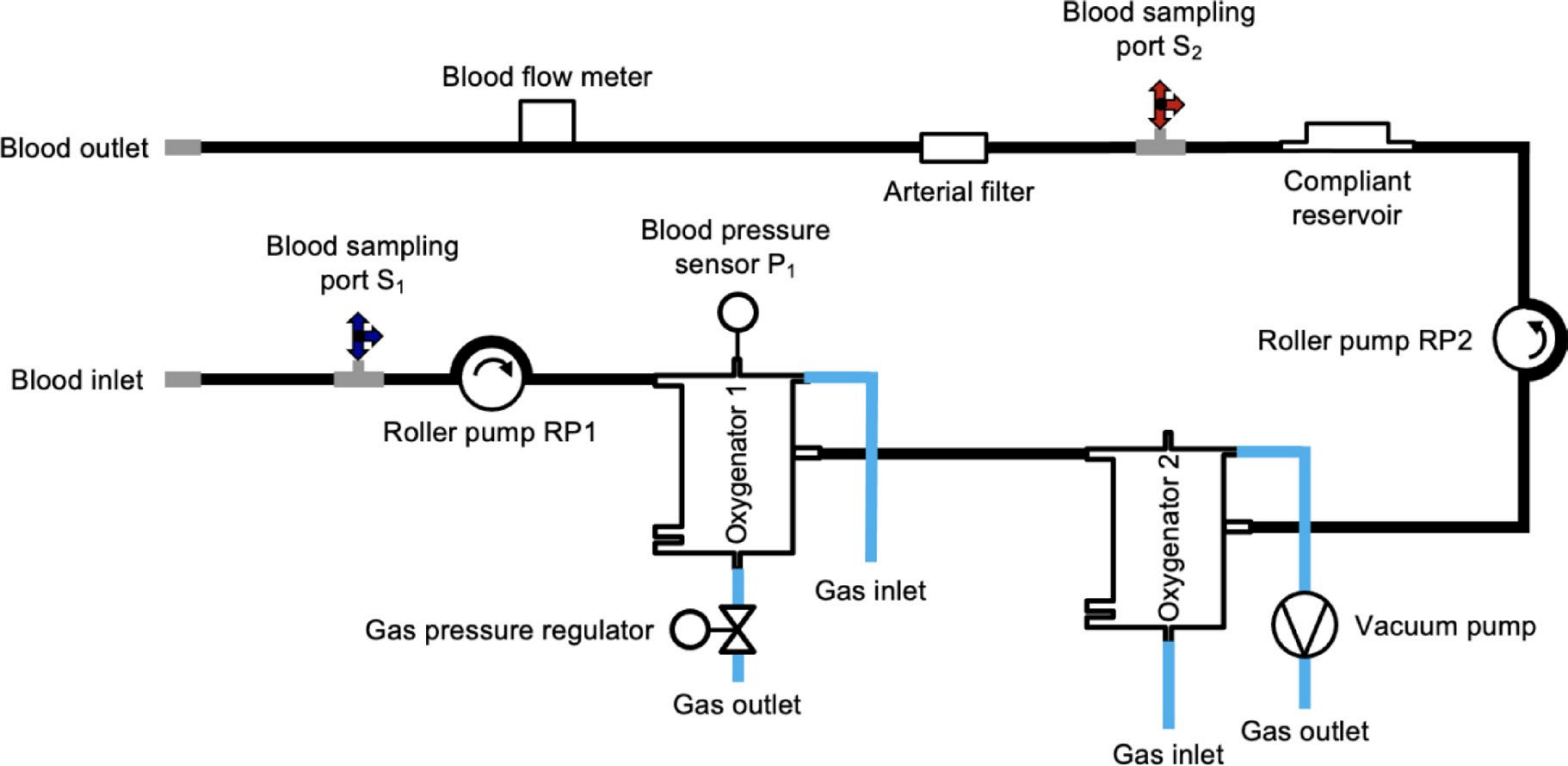
Schematic illustration of the cEHT system used for extracorporeal CO elimination. CO-poisoned blood circulates through two membrane oxygenators arranged in series. The first oxygenator receives high-pressure oxygen, generating a hyperoxic environment that facilitates CO dissociation from Hb; the released CO is removed via the gas outlet. A vacuum pump applies a negative pressure of approximately 400 mbar to the second oxygenator to deplete excess dissolved oxygen, ensuring a bubble-free transition during the pressure release at roller pump 2 (RP2). Blood pressure is increased above gas pressure in the first oxygenator by RP2 rotating at a lower speed than roller pump 1 (RP1), preventing oxygen permeation. Treated blood passes through a compliant reservoir and arterial filter before reentering circulation.

### The system for continuous extracorporeal hyperoxygenation therapy (cEHT): Design and function

The cEHT system is a custom-built extracorporeal circuit developed to enhance CO elimination by inducing a hyperoxic environment. A schematic illustration of the system is shown in Fig. 6, and a photograph of the in vitro setup is provided in Supplementary Fig. S1.

The circuit consists of two membrane oxygenators connected in series. Both oxygenators are modified by sealing the housings to enable a use at higher pressure. Blood flow through the system is maintained by two peristaltic roller pumps (roller pump 1 (RP1) and roller pump 2 (RP2)) adapted from the HL20 heart-lung machine (Maquet GmbH, Rastatt, Germany). The first pump (RP1) is positioned before the first oxygenator. The second pump (RP2), located after the second oxygenator, is set to a lower rotational speed than the first, thereby increasing pressure within the blood compartment of both oxygenators (measured at the blood pressure sensor P_1_). Simultaneously, a sweep gas flow at elevated gas pressure is supplied to the first oxygenator. The gas pressure is monitored and regulated by a pressure regulator. To avoid permeation of oxygen into blood, the gas pressure is always kept below the blood pressure. The resulting hyperoxic environment enhances oxygen transfer and increases oxygen solubility in the blood, thereby facilitating the dissociation of CO from Hb. The released CO is removed via the gas outlet of the first oxygenator. Due to the high oxygen partial pressure, gas bubbles would form within the blood, once the pressure is released. Therefore, excess oxygen is removed in the second oxygenator via a connection to a vacuum pump (LABOPORT N 96, KNF DAC GmbH, Hamburg, Germany). A compliant silicone reservoir (R-14, Medtronic Inc., Minneapolis, MN, USA) is placed after the second blood pump to reduce pressure pulsations at the outlet of the circuit. An arterial filter (Baby Sherlock, EUROSETS S.r.l., Medolla, Italy) is used to remove any microbubbles and particulate matter. The heat exchangers of both oxygenators are connected to a heater unit (HICO-AQUATHERM 660, pfm medical hico GmbH, Cologne, Germany) to maintain a blood temperature of 37 °C.

The blood temperature is measured at the corresponding port of the outlet of the first oxygenator. Blood flow rate is monitored in the return line, and blood samples for COHb analysis are collected at blood sampling ports S₁ and S₂. An ultrasonic bubble detector (BC100, GAMPT mbH, Merseburg, Germany) is used to continuously monitor for air emboli.

For the in vitro experiments, two types of oxygenators were used: the hilite 7000 (Xenios AG, Heilbronn, Germany), with a priming volume of 275 mL and a gas exchange surface area of 1.9 m²; and the hilite 800 (Xenios AG), with a priming volume of 55 mL and a surface area of 0.32 m². All blood flow rates, sweep gas flow rates, gas pressures, and blood compartment pressures for each experimental condition are detailed in Table 1. In vivo experiments were conducted using the hilite 7000 (Xenios AG, Heilbronn, Germany) for both the first and second oxygenator. The blood flow rate was set to 330 mL/min. The excess pressure within the blood compartment was between 3.6 and 4.0 bar and the excess gas pressure was maintained at 3.6 bar. The sweep gas flow supplied to the first oxygenator was 7 L/min and consisted of 95% oxygen and 5% carbon dioxide to prevent respiratory alkalosis. The second oxygenator was supplied with ambient air.

### In vitro experiments

As previously described^36^, fresh pooled porcine blood was obtained on the day of the experiment from a local slaughterhouse and fully anticoagulated with heparin with a target activating clotting time (ACT) of at least 150 s. A cardiopulmonary bypass machine (HL-20, Maquet, Rastatt, Germany) circulated blood through a closed-loop system consisting of a cardiotomy reservoir and a hilite 7000 (Xenios AG, Heilbronn, Germany). The blood was preconditioned according to ISO 7199^47^. To induce CO poisoning, a sweep gas mixture containing 3% carbon monoxide and 97% nitrogen (Linde AG, Pullach, Germany) was supplied to the gas compartment of the first oxygenator until the target COHb concentration of 40-45% was reached.

To assess the effect on CO elimination, membrane surface area, blood flow rate, gas flow rate, and sweep gas composition was varied in each case, while the other parameters remained unchanged (Table 1). After each experiment, blood was collected via the blood outlet after the second oxygenator, and COHb levels were analyzed by a blood gas analyzer (ABL800 Flex, Radiometer Medical ApS, Brønshøj, Denmark) to assess the efficiency of CO elimination. Each setup was performed three times using freshly CO-poisoned porcine blood. Blood temperature was maintained at 37 °C.

### In vivo study protocol

**Fig. 7:**
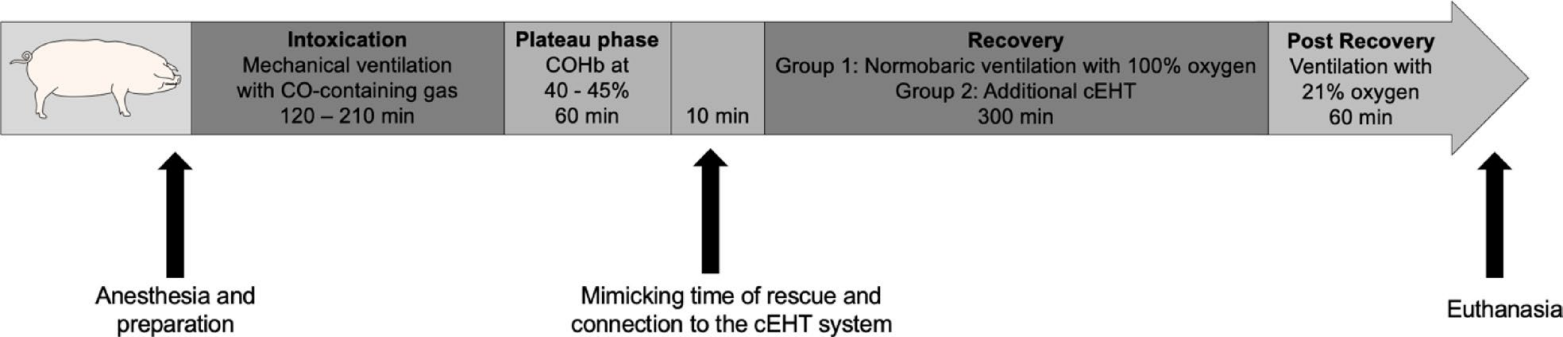
Experimental timeline of in vivo experiments.

All animal experiments were conducted in compliance with institutional and national regulations, adhered to the principles of laboratory animal care, and followed the ARRIVE guidelines. The experimental protocol was approved by the governmental animal care committee (Landesamt für Verbraucherschutz und Ernährung Nordrhein-Westfalen, Recklinghausen, Germany). Six female German Landrace pigs with a mean body weight of 79.2 ± 6.2 kg were included. The study protocol for the in vivo experiments was performed as previously described^36^(Fig. 7). Briefly, all animals were admitted to the facility two weeks before the experiment for acclimatization and veterinary evaluation. Twelve hours prior to the intervention, food was withheld while water remained freely available. Premedication was performed intramuscularly using 1 mL atropine (1%), 4 mg/kg azaperone, and 10 mg/kg ketamine. Following placement of an intravenous line in the ear vein, the animals were intubated and anesthesia was maintained with propofol (5-10 mg/kg/h) and fentanyl (820 µg/kg as a one-time bolus). Crystalloid fluids were administered at 1 mL/kg/h, and a urinary catheter was inserted for monitoring.

Mechanical ventilation was adjusted to maintain a target p_a_CO₂ of 35-45 mmHg, with a positive end-expiratory pressure (PEEP) of 5 mbar, respiratory rate of 10-18 breaths per minute, and a tidal volume of 6-10 mL/kg. Arterial access was obtained via a femoral artery catheter for continuous blood pressure monitoring and blood sampling. A 13 Fr high-flow double-lumen dialysis catheter and a four-lumen central venous catheter were inserted into the femoral veins for cEHT connection and medication administration. A pulmonary artery catheter was placed via the right internal jugular vein to continuously monitor pulmonary artery pressure and intermittently assess pulmonary capillary wedge pressure, cardiac output, and to allow for additional blood sampling.

Continuous heparin infusion was initiated to maintain an activated clotting time above 150 s; boluses were given as needed to achieve the target anticoagulation levels. Body temperature was maintained at 37 °C by using the heat exchanger of both oxygenators as well as by using an air warming system (Bair Hugger, 3 M Deutschland), when needed.

The experimental timeline consisted of five phases: CO intoxication, plateau, rescue, recovery, and post-recovery. For CO exposure (first phase), a gas mixture containing 940 ppm CO, 20% oxygen, and nitrogen balance was delivered via the ventilator, at a fractional inspired oxygen concentration (FiO₂) of 0.21. Arterial blood samples were collected every 15 min to monitor the increase in COHb until target levels of 40-45% were reached. In the subsequent plateau phase, COHb was maintained within this range for 60 min by adjusting the inspired gas mixture (plateau phase, 2nd phase). Blood gases were taken every 10 min to ensure stable COHb levels.

Both groups then underwent a 10-minute ventilation phase with room air (FiO₂ = 0.21) to simulate the delay between CO exposure and the initiation of emergency treatment. At the beginning of this phase, a 25,000 IU heparin bolus was administered. Thereafter the animals were randomly assigned to two treatment groups, control and cEHT. During the subsequent recovery phase, the control group received ventilation with 100% oxygen for 300 min through the tracheal tube without being connected to the cEHT system. The animals in the cEHT group received treatment through the cEHT system in addition to the ventilation with 100% oxygen. The cEHT system was connected via the dialysis catheter previously placed in the femoral vein. Prior to connection, the tubing and outer compartment of the cEHT system were primed with 0.9% saline to compensate for the extracorporeal volume. The treatment with the cEHT system was continued for 75 min or until COHb levels fell below 5%. The ventilation was continued until the end of the recovery phase. Blood samples were taken every 15 min.

An additional 60-minute post-recovery phase followed, during which animals were ventilated with room air (FiO₂ = 0.21). An additional 60-minute post-recovery phase followed, during which animals were ventilated with room air (FiO_2_ = 0.21). During this phase, arterial blood gas analyses were performed every 15 min to determine COHb, pO_2_, pCO_2_, and lactate levels, while plasma-free Hb was assessed every 60 min.

During the whole experiment, vital signs-including core temperature, heart rate, arterial and central venous pressure, pulmonary artery pressure, and central venous oxygen saturation-were continuously recorded at 0.2 Hz. Additional parameters such as respiratory rate, minute ventilation, FiO₂, end-tidal oxygen concentration, and drug administration were documented every 15 min.

At the end of the experiment, tissue samples from the heart and brain were collected for histopathological evaluation.

### Histological processing and immunohistochemistry

Histological processing was performed according to the protocol described by Li et al.^48^. Tissue samples from the heart and brain were collected post-mortem and fixed in 10% neutral-buffered formalin for 24 h at room temperature. Following fixation, tissues were dehydrated through a graded ethanol series, cleared in xylene, and embedded in paraffin. Serial sections of 4 μm thickness were cut using a microtome and mounted on glass slides. For general histological assessment, sections were deparaffinized, rehydrated, and stained with hematoxylin and eosin (HE staining) to evaluate tissue morphology and identify potential ischemic lesions. Microscopic evaluation was conducted using a light microscope (BX43, Olympus, Tokyo, Japan), and images were captured with a digital camera system (SC180, Olympus).

### Assessment of hemolysis

Hemolysis was assessed by determining the concentration of plasma free Hb via photometric analysis (Ultrospec 2100 Pro, Biochrom, Berlin, Germany). Measurements were performed following the cyanmethemoglobin method (Hemoglobin FS, DiaSys, Germany) in accordance with DIN 58931:2021-09^49^ and the manufacturer’s instructions. Plasma separation was achieved by centrifuging samples twice at 1,500 x g for 15 min.

### Analyses and statistics

The primary outcome parameter was the percentage of carboxyhemoglobin (COHb), which was measured using a blood gas analyzer (ABL800 Flex, Radiometer Medical ApS, Brønshøj, Denmark).

The COHb half-life was determined by nonlinear regression using a one-phase exponential decay model with the plateau set to 0% COHb. Prior to all statistical comparisons, data were tested for normality using the Shapiro-Wilk test. The majority of the variables followed a normal distribution. However, COHb levels over time (Fig. 2b) and the parameters presented in Supplemental Figs. 1a-c were identified as non-normally distributed. For comparisons between two groups, either an unpaired two-tailed t-test (for normally distributed data) or the Mann-Whitney U test (for non-normally distributed data) was applied. For longitudinal data involving multiple time points, a two-way ANOVA was used. Continuous variables were assessed for normality using the Shapiro-Wilk test. Due to the small sample size (*n* = 3) per groupx, non-normally distributed data are presented as median and range [minimum; maximum]. Normally distributed data are reported as mean ± standard deviation (SD). Statistical analyses were performed using GraphPad Prism 9 (GraphPad Software, San Diego, CA, USA). For comparisons between two groups of non-normally distributed data, the Mann-Whitney U test was applied. Statistical analyses were performed using GraphPad Prism 9 (GraphPad Software, San Diego, CA, USA). A two-way ANOVA with Geisser Greenhouse correction was used to compare curves. Multiple comparison was compared using the Fisher`s LSD test.

## Supplementary Information

Below is the link to the electronic supplementary material.

Supplementary Material 1

Supplementary Material 2

## Data Availability

All data generated or analyzed during this study are included in the published article and its supplementary materials. Additional data are available from the corresponding author upon reasonable request.

## Declarations

### Humane endpoints

All animals were kept under continuous deep anesthesia throughout the entire experiment and were euthanized at the planned end of the protocol. As this study was conducted as a non-survival model, the animals never regained consciousness and therefore additional humane endpoints were not applicable. The protocol was approved by the responsible institutional animal care and use committee and was conducted in full compliance with all relevant ethical guidelines.
