# Supplementary figures and images for: Continuous extracorporeal hyperoxygenation therapy reduces carbon monoxide half-life time in a carbon monoxide-poisoned pig model: a feasibility study
Source: Sci Rep. 2026 Jul 2;16:20351. doi: 10.1038/s41598-026-57491-5 (PMC13328516; doi:10.1038/s41598-026-57491-5)

**Supplements**


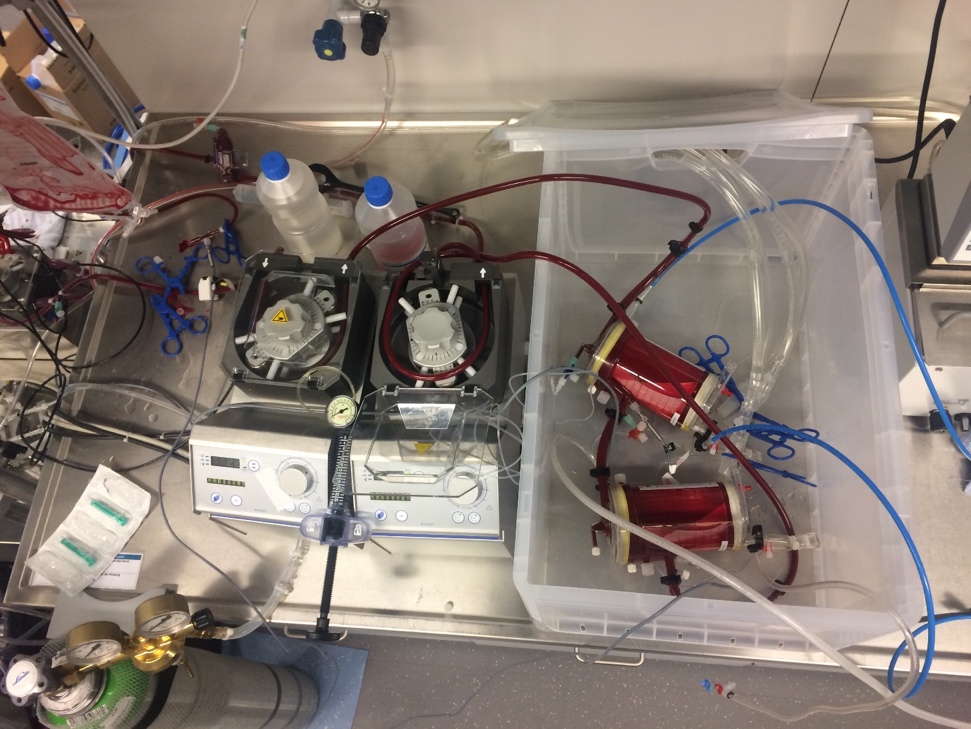


**Fig. S1: Setup of the cEHT system within the in vitro circulation circuit.**

Supplement: Supplementary file 1 — Supplementary Material 1 [file 41598_2026_57491_MOESM1_ESM.docx]
