# Supplementary material for: Continuous extracorporeal hyperoxygenation therapy reduces carbon monoxide half-life time in a carbon monoxide-poisoned pig model: a feasibility study
Source: Sci Rep. 2026 Jul 2;16:20351. doi: 10.1038/s41598-026-57491-5 (PMC13328516; doi:10.1038/s41598-026-57491-5)
Supplement: Supplementary file 2 — Supplementary Material 2 [file 41598_2026_57491_MOESM2_ESM.docx]

**Supplementary Figure S2**


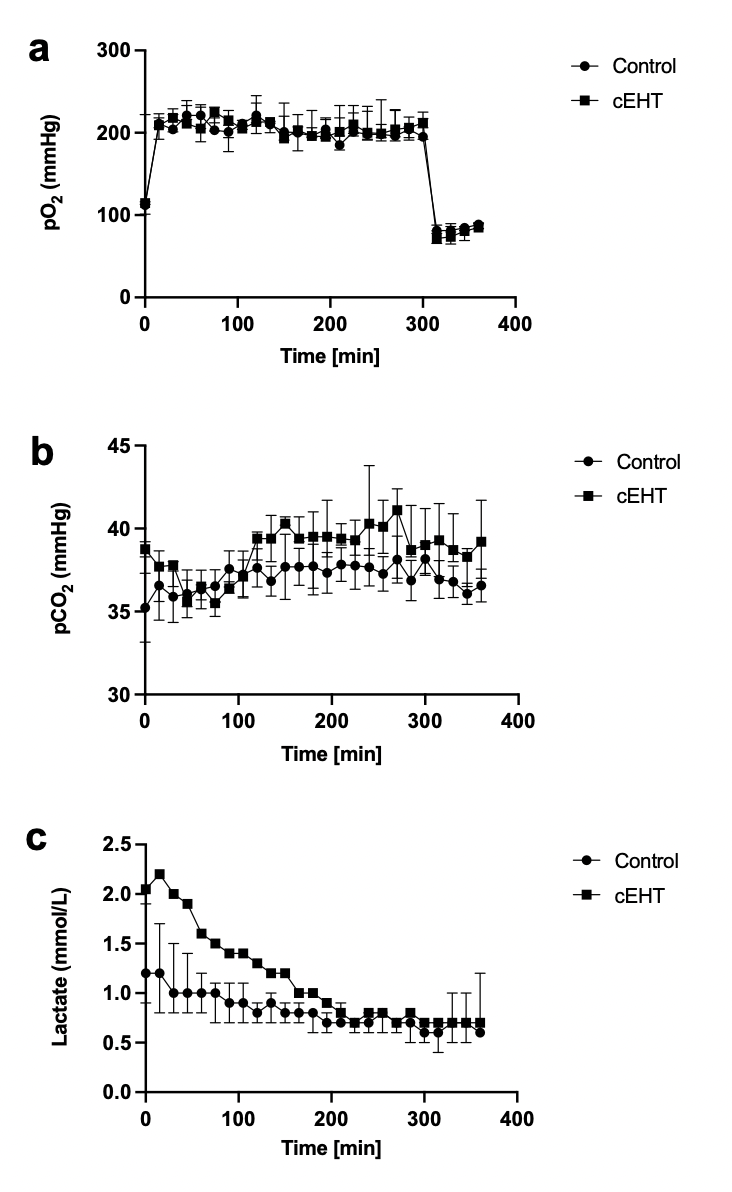


**Supplementary Figure S2.** Time course of arterial (a) pO_2_ , (b) pCO_2_ , and (c) lactate levels in the control group and the cEHT group. Data are presented as median with range. No significant differences were observed between the groups during the treatment period.
